# Supplementary material for: A method for evaluating sediment-induced macroinvertebrate community composition changes in Idaho streams
Source: PeerJ. 2024 Oct 4;12:e18060. doi: 10.7717/peerj.18060 (PMC11457874; doi:10.7717/peerj.18060)
Supplement: Supplemental Information 1 [file peerj-12-18060-s001.docx]

**Site Class Descriptions**

**Table S1.** Site class descriptions.

| **Site Class** | **Level 4 Ecoregions** |
| --- | --- |
| Mountains | The Wasatch-Unita mountains and selected sub-ecoregions of the Northern Rockies, Idaho Batholith, and the Middle Rockies (15i, 15o, 15p, 15y, 16 (except 16f), 17ad, 173, 19) |
| Foothills | The Blue mountains and selected sub-ecoregions of the Northern Rockies and Middle Rockies (11, 15j, 15f, 15m, 15n, 15s, 15u, 15v, 15w, 170, 17ab, 17j) |
| Plains Plateaus and Broad Valleys (PPBV) | The Columbia Plateau, Northern Basin and Range, Central Basin and Range, Snake River Plain, Wyoming Basin and selected sub-ecoregions of the Middle Rockies and Idaho Batholith (10, 12, 13, 17aa, 17n, 18, 80). |

Site classes development is described in detail in Jessup (2011) and also in Jessup and Pappani (2015) and DEQ (2016).

**Physical Variable Relationships to % fines < 2.5 mm (BURP data)**

| **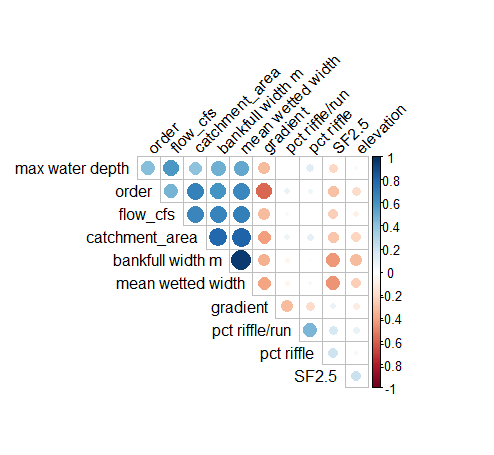**  **Figure S1.** BURP reference site correlations. Size and color indicate coefficient magnitude. | **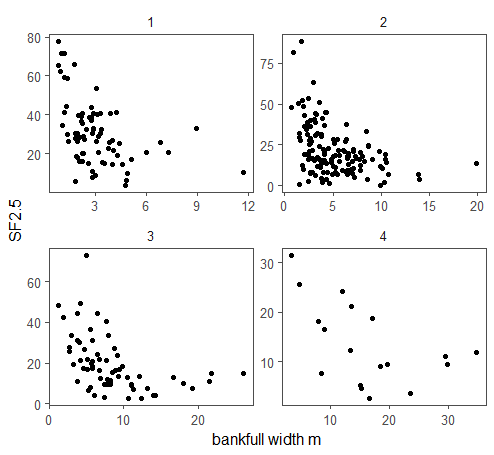**  **Figure S2**. Relationship between bankfull width and % fines < 2.5 mm among BURP reference sites by stream order. |
| --- | --- |
| **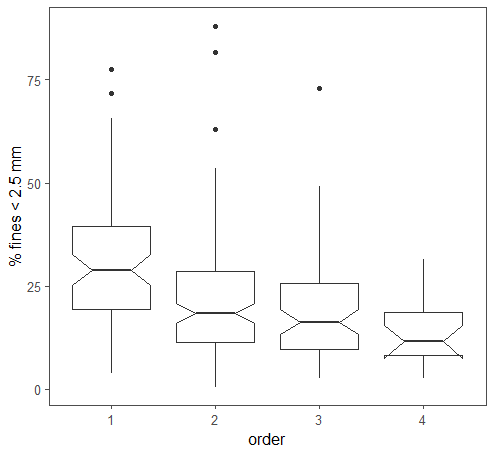**  **Figure S3.** Relationship between stream order and % fines < 2.5 mm among BURP reference sites. |  |

**Table S2**. Comparison of BURP, PIBO, and AIM monitoring programs.

|  | **BURP** | **PIBO** | **AIM** |
| --- | --- | --- | --- |
| Agency | DEQ | USFS | BLM |
| Stream types sampled | wadable 1^st^-4^th^ order | wadable 1^st^-4^th^ order streams with < 4% gradient with and ≥ 50% federal ownership | Wadable 1^st^-4^th^ order streams, and some large rivers using a boat-based protocol |
| Coverage | Idaho streams outside wilderness, including private and public lands | USFS and BLM lands | BLM lands |
| Sample design | Targeted nonrandom | Rotating panel survey design, plus some targeted nonrandom sites | Probability survey design, plus some targeted nonrandom sites |
| Sample timing | Summer baseflow | Summer baseflow | Summer baseflow |
| Repeat sampling of same reach across years | No | Yes - most sites sampled every 5 years, a subset sampled annually or biennially | Yes - most sites sampled every 5 years |
| Reference sites definition | Among 1998-2007 BURP sites, 10% least-disturbed sites based on 9 different land use criteria (Jessup and Pappani 2015). | Sites within Wilderness or meet all of: no obvious mining, no grazing within 30 years, < 5% timber harvest, road density < 0.5 km/km^2^ | No program-specific reference sites |
| Reach delineation | 30 x bankfull width, 100 m minimum | 20 x bankfull width, 160 m min and 500 m max length. | 20 x bankfull width, 150 m min, 4 km max |
| Pebble count | Modified Wolman method, % < 2.5 mm, based on at least 50 particles from each of 3 riffle transects | Modified Wolman method, % < 2mm, based on 5 particles from each of 20 transects within reach | Modified Wolman method, % < 2 mm, based on 10 particles from each of 21 transects within reach |
| Macroinvertebrate sample collection | Composite of 3 500 µm mesh Hess samples from 3 riffle habitats | Composite of 8 500 µm mesh Surber net samples from riffle habitats | Composite of 8 500 µm mesh Surber net samples from riffle habitats or of 11 Surber samples spanning multiple habitat types |
| Taxonomic laboratory | EcoAnalysts | Utah State University / National Aquatic Monitoring Center | Utah State University / National Aquatic Monitoring Center |
| Macroinvertebrate condition assessment approach | MMI (SMI2 ≥ 2) | PIBO RIVPACS O/E model; target O/E > 0.78 | PIBO RIVPACS O/E model; target O/E > 0.63 |
| FSBI calculation | Yes. Calculated for each BURP sample 1998-present. See methods in supporting materials. | Not calculated. Calculated for this study. | Not calculated. Calculated for this study. |
| Data used in this study | 1998-2007 for reference benchmarks; 1998-2021 for stressor-response benchmarks and framework performance analysis. | 1^st^-4^th^ order reaches sampled by PIBO in Idaho 2004-2019 | 1^st^-4^th^ order reaches sampled by AIM in Idaho 2013-2022. |

**Logistic Regression Curves**

| 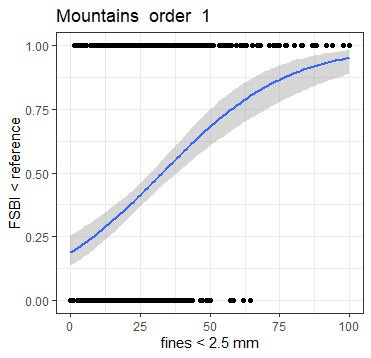 | 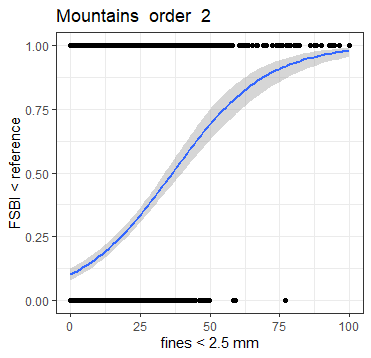 |
| --- | --- |
| 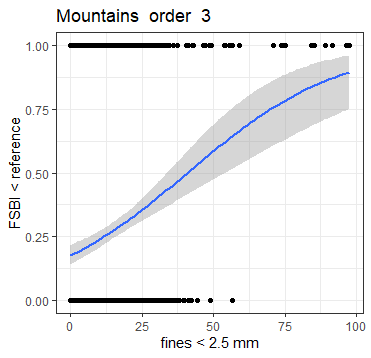 | 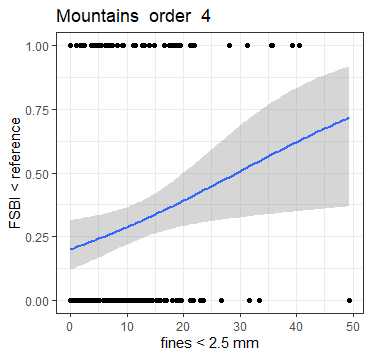 |

**Figure S1**. Stressor-response logistic regressions for the Mountains site class.

| 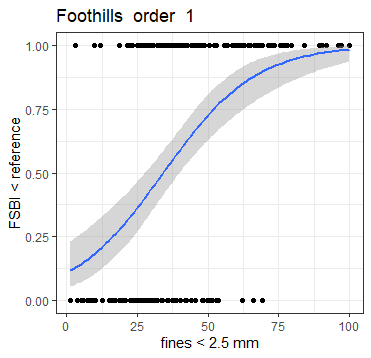 | 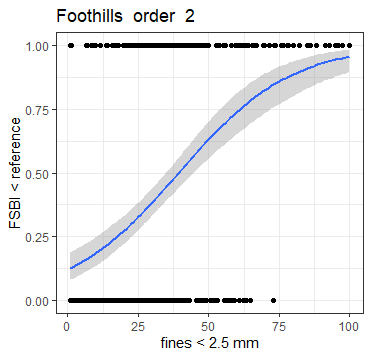 |
| --- | --- |
| 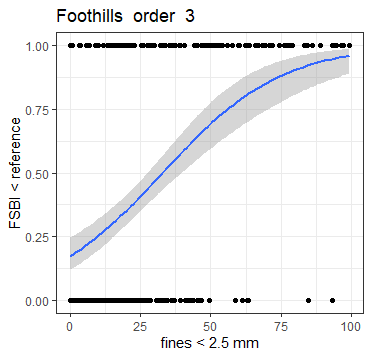 | 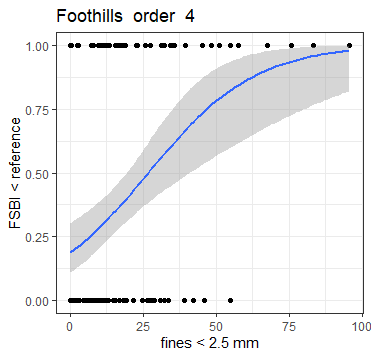 |

**Figure S2**. Stressor-response logistic regressions for the Foothills site class.

| 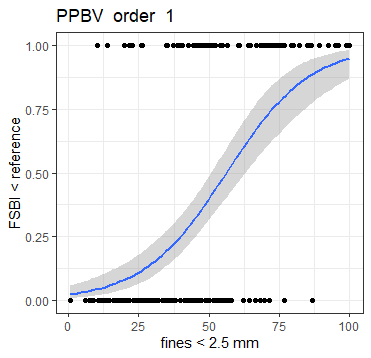 | 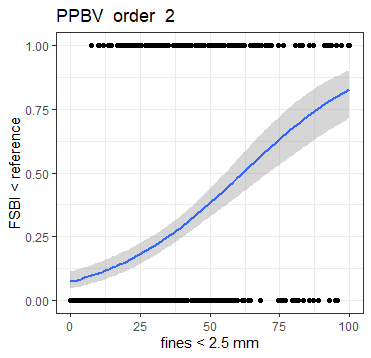 |
| --- | --- |
| 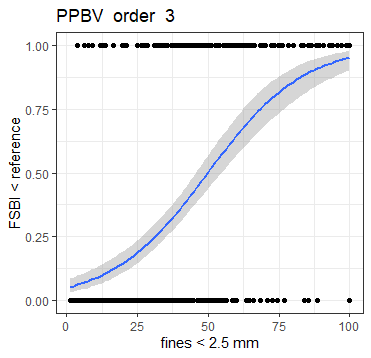 | 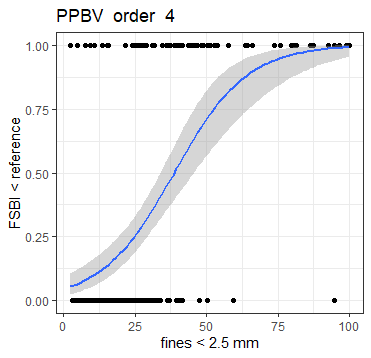 |

**Figure S3**. Stressor-response logistic regressions for the Plains Plateaus and Broad Valleys (PPBV) site class.

**FSBI Calculation Methods**

*BURP data*

The taxonomic laboratory (EcoAnalysts) identified and counted all individuals within each macroinvertebrate sample to the lowest possible taxonomic resolution, usually to genus or species. DEQ then assigned each taxa reported by the laboratory to an operational taxonomic unit (called a ‘validated taxa’ by DEQ) prior to importing macroinvertebrate data in to DEQ’s database. Validated taxa are those for which have taxa attributes (including a FSBI-level taxa score) in DEQ’s database and were used for development of BURP MMIs. In cases where a taxa identified by the laboratory is not in DEQ’s validated taxa table, DEQ staff either add the taxa and its attributes to the database, or assign the taxa to a higher level validated taxa if reasonable. For example, the laboratory reports *Example species* in results, but this species is not in the database but a record for the associated genus is, in some cases the taxonomic result was imported as *Example sp.* if DEQ staff determined attributes were similar. Sample FSBI was calculated as the sum of all taxa-level FSBI scores associated with validated taxa within a sample.

*PIBO and AIM data*

For the PIBO and AIM programs, the taxonomic laboratory (National Aquatic Monitoring Center at Utah State University) identified up to 600 individual specimens from each sample to the lowest possible taxonomic resolution, usually to genus or species. Raw laboratory taxonomic identifications were then assigned to an operational taxonomic unit used for purposes of O/E calculations. USFS and BLM then randomly subsampled identified taxa to create a list of 300 identified individuals for each sample.

At the time of this study, the PIBO and AIM programs did not calculate sample FSBI from their macroinvertebrate data. USFS and BLM provided harmonized subsampled macroinvertebrate data to DEQ for FSBI calculations. Based on Relyea et al. 2012, DEQ assigned a taxa-level FSBI score to each unique taxa name reported by the laboratory (ScientificName values) included in subsampled data. Sample FSBI scores were then calculated using an R tool developed for this project (<https://github.com/jjwill2/FSBI_calculator>). Sample FSBI scores were calculated as the sum of assigned taxa-level FSBI scores within a sample. Assigned taxa-level FSBI scores and R inputs and outputs are available through the github link.

**Table S4.** Fine sediment benchmarks reported in the literature and used for comparison to calculated benchmarks. All thresholds are for surface fine sediment, except where noted otherwise.

| **Source** | **Benchmark Description** | **Biological Receptor** | **Sediment**  **Size Fraction** | **Stream Size** | **Threshold**  **Value** |
| --- | --- | --- | --- | --- | --- |
| Bryce et al. 2010 | Minimum effects threshold for Index of Biotic Integrity (IBI) value decreases in wadable Western U.S. mountain streams | aquatic vertebrates | ≤ 0.06 mm | wadable | 5% |
|  |  | aquatic vertebrates | ≤ 2 mm | wadable | 13% |
|  |  | aquatic macroinvertebrates | ≤ 0.06 mm | wadable | 3% |
|  |  | aquatic macroinvertebrates | ≤ 2 mm | wadable | 10% |
| Jessup et al. 2014 | Recommended reference benchmark for New Mexico streams in mountains site class, based on statistical distribution within reference sites and changepoint analysis for multiple biological indicators. | aquatic macroinvertebrates | ≤ 2 mm | wadable | 20% |
| Reylea et al. 2012 | Percent fines levels at which sediment-sensitive macroinvertebrate taxa start disappearing from northwest US streams. | aquatic macroinvertebrates | < 2 mm | 1-6^th^ order | 10-20% |
| Jensen et al. 2009 | Surface plus subsurface fine sediment threshold for rapid decreases of egg-to-fry survival, based on a meta-analysis of lab and field studies in peer-reviewed literature | Chinook, Coho, and Chum salmon, and steelhead trout | < 0.85 mm | NA (lab & field experiments) | 10% |
| Jensen et al. 2009 | Surface plus subsurface fine sediment threshold for rapid decreases of egg-to-fry survival, based on a meta-analysis of lab and field studies in peer-reviewed literature | Chinook, Coho, and Chum salmon, and steelhead trout | < 3.4 mm, < 4.8 mm, < 6.4 mm | NA (lab & field experiments | 25-30% |
| Burdon et al. 2013 | Threshold above which rapid changes in pollution-sensitive invertebrates (% Ephemeroptera, Plecoptera, and Trichiptera) were observed across 20 New Zealand agricultural streams. | Aquatic macroinvertebrates | < 2 mm | 1-3^rd^ order | 20% |
| EPA 2020 (Table 4.2) | Threshold used to identify reference sites within the Northern Rockies and Pacific Mountains region for the National River and Streams Assessment (NRSA) | NA (reference statistical distribution) | unclear | wadable & non-wadable | 15% |
| EPA 2020 (Table 4.2) | Threshold used to identify most-disturbed sites within the Northern Rockies and Pacific Mountains region for the National River and Streams Assessment (NRSA) | NA (reference statistical distribution) | unclear | wadable & non-wadable | 50% |
| EPA 2007 | ‘Good’ condition threshold used by EPA for assessing stream conditions in EMAP Report ‘Ecological Condition of Wadable Streams of the Interior Columbia River Basin’. ‘Good’ percent fines condition was calculated as 75^th^ percentile value among least-disturbed reference sites within Northern Rockies, Idaho Batholith, Middle Rockies, and Canadian Rockies ecoregions. | NA (reference statistical distribution) | < 2 mm | wadable | 16.19% |
| EPA 2007 | ‘Good’ condition threshold used by EPA for assessing stream conditions in EMAP Report ‘Ecological Condition of Wadable Streams of the Interior Columbia River Basin’. ‘Good’ percent fines condition was calculated as 75^th^ percentile value among least-disturbed reference sites within Columbia Plateau and Northern Basin & Range ecoregions. | NA (reference statistical distribution) | < 2 mm | wadable | 29.52% |
| Miller et al., 2021 | 70^th^ percentile value among Northwest U.S. stream reference sites within Northern Rockies level III ecoregion | NA (reference statistical distribution) | < 2 mm | ≤10 m bankfull width | 29% |
| Miller et al., 2021 | 70^th^ percentile value among Northwest U.S. stream reference sites within Northern Rockies level III ecoregion | NA (reference statistical distribution) | < 2 mm | > 10 m bankfull width | 15% |
| Miller et al., 2021 | 70^th^ percentile value among Northwest U.S. stream reference sites within Xeric Basins level III ecoregion | NA (reference statistical distribution) | < 2 mm | ≤10 m bankfull width | 45% |
| Miller et al., 2021 | 70^th^ percentile value among Northwest U.S. stream reference sites within Xeric Basins level III ecoregion | NA (reference statistical distribution) | < 2 mm | > 10 m bankfull width | 44% |
| USFS et al. 1997 | Surface fine sediment levels recommended by USFS and BLM in their draft EIS for Upper Columbia River Basin developed. Recommended values vary based on Rosgen channel type (A-C) and geology (plutonic, volcanic, or metamorphic rock). See also DEQ 2003. | Anadromous salmonids | < 6 mm | unclear | 14-37% |
| USFS 1995 | PacFish/InFish Environmental Assessment Alternative E (‘which was not ultimately selected) stated “A Riparian Management Objective for sediment substrate would be established to be less than 20 percent fine sediment in spawning habitat” (USDA 1995, pA-16) | Anadromous salmonids | unclear | unclear | 20% |
| CRITFC 1995. | Recommended surface fine sediment levels for an in-channel habitat condition coarse screening process. In cases where the benchmark is exceeded, land management actions to reduce sediment were recommended. | Anadromous salmonids | < 6 mm | unclear | 20% |
| USFS et al. 1998 | Recommended surface fines benchmark for use in making Endangered Species Act determinations of effect for individual or group actions at the watershed scale for the Clearwater Basin and Lower Salmon, applies to Rosgen channel type A & B | Chinook, Steelhead, and Bull Trout | < 6 mm | Not specified | ≤10% |
| USFS et al. 1998 | Recommended surface fines benchmark for use in making Endangered Species Act determinations of effect for individual or group actions at the watershed scale for the Clearwater Basin and Lower Salmon, applies to Rosgen channel type C & E | Chinook, Steelhead, and Bull Trout | < 6 mm | Not specified | ≤20% |
| NMFS 1996 | Recommended percent fines in gravel for streams classified as ‘properly’ functioning for purposes of making Endangered Species Act determinations of effect | Anadramous salmonids | < 0.85 | Not specified | < 12% |

**Works Cited**

Bryce, S.A., Lomnicky, G.A., Kaufmann, P.R. 2010. Protecting sediment-sensitive aquatic species in mountain streams through the application of biologically based streambed sediment criteria. *Journal of the North American Benthological Society*. 29(2): 657-672.

Burdon, F., McIntosh, A.R., Harding. J.W. 2013. Habitat loss drives threshold response of benthic invertebrate communities to deposited sediment in agricultural streams. Ecological Applications. 23(5): 1036-1047.

CRITFC (Columbia River Inter-Tribal Fish Commission). 1992. Wy-Kan-Ush-Mi_Wa_kish-Wit (Spirit of the Salmon). The Columbia River Anadromous Fish Restoration Plan of the Nez Perce, Umatilla, Warm Springs, and Yakama Tribes, Portland, OR.

DEQ 2003. Guidance to selection of sediment targets for use in Idaho TMDLs. June 2003. https://www2.deq.idaho.gov/admin/LEIA/api/document/download/4843

DEQ. 2016. Idaho Water Body Assessment Guidance, 3^rd^ edition.

EPA, 2007. Ecological condition of wadable streams of the interior Columbia River Basin. EPA-910-R-07-005. US Environmental Protection Agency, Region 10, Seattle, Washington. <https://archive.epa.gov/emap/archive-emap/web/pdf/icbfinal07.pdf>

EPA, 2020. National Rives and Streams Assessment 2013-2014 Technical Support Document. Office of Water. Washington DC. EPA 843-R-19-002.

Jensen, D.W., Steel, E.A., Fullerton, A.H., Pess, G.R. 2009. Impact of fine sediment on egg-to-fry survival of pacific salmon: a meta-analysis of published studies. *Reviews in Fisheries Science* 17(3): 348-359.

Jessup, B. 2011. Biological Assessment Frameworks and Index Development for Rivers and Streams in Idaho. Owings Mills, MD.: TetraTech, Inc.

Jessup, B.K., Kaufman, P.R., Forrest, J., Guevara, L.S., Joseph, S. 2014. Bedded sediment conditions and macroinvertebrate responses in New Mexico streams: a first step in establishing sediment criteria. *Journal of the North American Benthological Society*. 50(6): 1558-1574.

Jessup, B., Pappani, J. 2015. Combination of biological and habitat indices for assessment of Idaho streams. *Journal of the American Water Resources Association*. 51(5): 1408-1417.

Miller, S.W., N. Cappuccio, J. Courtwright, L. Littlejohn, B. Bohn. 2021. A Statewide Assessment of BLM-Managed Streams and Rivers in Idaho. U.S. Department of the Interior, Bureau of Land Management, National Operations Center, Lakewood, Colorado.

Relyea, C., Minshall, G.W., Danehy, R.J. 2012. Development and validation of an aquatic fine sediment biotic index. *Environmental Management* 49: 242-252.

U. S. Forest Service and Bureau of Land Management. 1997. Interior Columbia Basin Ecosystem Management Project (ICBEM). Upper Columbia River basin draft environmental impact statement. Boise, ID.

USDA-FS, USDI-BLM, NOAA Fisheries, and U.S. Fish and Wildlife Service (North Central Idaho Level 1 Team). 1998. Matrix of pathways and indicators of watershed conditions for Chinook salmon, steelhead trout, and bull trout - local adaptation for portions of the Clearwater Basin and Lower Salmon. U. S. Forest Service, Clearwater National Forest, Nez Perce National Forest, and Bitterroot National Forest, and Bureau of Land Management, Cottonwood Field Office, Orofino and Grangeville, ID, Hamilton, MT, and Cottonwood, ID.

U.S. Forest Service, 1995. Inland Native Fish Strategy Environmental Assessment Decision Notice and Finding of No Significant Impact. Intermountain, Northern, and Pacific West Regions.

National Marine Fisheries Service (NMFS), 1996. Making endangered species act determinations of effect for individual or grouped actions at the watershed scale. Augus 1996. NMFS Environmental and Technical Services Division. Habitat Conservation Branch.
